# Risk factors for the carriage of *Streptococcus infantarius* subspecies *infantarius* isolated from African fermented dairy products

**DOI:** 10.1371/journal.pone.0225452

**Published:** 2019-11-27

**Authors:** Kossia D. T. Gboko, Sylvain G. Traoré, Aimé R. Sanhoun, Jérôme Kirioua, Nize Otaru, Fabienne Kurt, Fabienne N. Jaeger, Julia Isenring, Dasel W. M. Kaindi, Bernd Kreikemeyer, Pierre Renault, Jan Hattendorf, Leo Meile, Christoph Jans, Roland Nguetta, Bassirou Bonfoh

**Affiliations:** 1 Centre Suisse de Recherches Scientifiques en Côte d’Ivoire (CSRS), Adiopodoumé Yopougon, Abidjan, Côte d'Ivoire; 2 University of Peleforo Gon Coulibaly, Korhogo, Côte d'Ivoire; 3 University of Nangui Abrogoua, Abidjan, Côte d'Ivoire; 4 Laboratory of Food Biotechnology, Institute of Food, Nutrition and Health, ETH Zurich, Schmelzbergstrasse 7, Zurich, Switzerland; 5 Department of Epidemiology and Public Health, Swiss Tropical and Public Health Institute, Basel, Switzerland; 6 University of Basel, Basel, Switzerland; 7 Department of Food Science, Nutrition and Technology, University of Nairobi, Nairobi, Kenya; 8 Institute of Medical Microbiology, Virology, and Hygiene, Rostock University Medical Centre Rostock, Germany; 9 Institut National de la Recherche Agronomique, UMR 1319 MICALIS, Jouy-en-Josas, France; 10 Institut de cardiologie d’Abidjan (ICA), Abidjan, Côte d’Ivoire; 11 Université Felix Houphouët-Boigny, Abidjan, Côte d’Ivoire; Hitit University, TURKEY

## Abstract

*Streptococcus infantarius* subsp. *infantarius* (*Sii*) has been identified as predominant lactic acid bacteria in spontaneously fermented dairy products (FDPs) in sub-Saharan Africa including Côte d'Ivoire. However, *Sii* belongs to the *Streptococcus bovis*/*Streptococcus equinus* complex (SBSEC). Most SBSEC members are assumed to be involved as opportunistic pathogens in serious diseases in both humans and animals. A population-based cross-sectional survey, including 385 participants was conducted in Korhogo, northern Côte d'Ivoire, to identify risk factors for *Sii* fecal carriage, including consumption of local FDPs. A structured questionnaire was used to gather participant's socio-demographic and economic characteristics, their relation to livestock and dietary habits. In addition, fresh stool and milk samples were collected. The identification of *Sii* was done using a SBSEC-specific PCR assay targeting 16S rRNA and *groEL* genes. The overall prevalence of SBSEC and *Sii* carriage was 23.2% (confidence interval CI 95% = 18.9–27.5) and 12.0% (CI 95% = 8.4–15.5) for stool, respectively. Prevalence of *Sii* was significantly higher in consumers of artisanal butter compared with non-consumers (57.1% vs 10.1%, odds ratio OR: 11.9, 95% CI: 3.9–36.6), as well as in persons handling livestock (OR = 3.9; 95% CI = 1.6–9.3) and livestock primary products (OR = 5.7; 95% CI = 2.3–14.3). The closer contact with livestock was a risk factor for *Sii* fecal carriage. *Sii* strains were isolated from fresh and fermented milk products with a prevalence of 30.4% and 45.4%, respectively. Analysis of *Sii* population structure through the SBSEC multi locus sequence typing assay revealed a close relationship across human and dairy isolates, possibly linked to a Kenyan human isolate. All these outcomes underline the interest of in-depth investigations on the ecology, potential reservoirs and pathways of contamination by *Sii* at the human-animal-environment interface in comparison to yet to be collected data from Europe, Asia and the Americas to further elucidate the various roles of *Sii*.

## Introduction

The *Streptococcus bovis/ Streptococcus equinus* complex (SBSEC) is a heterogeneous group of bacteria within Lancefield group D streptococci [1]. Based on genetic analyses, members of the SBSEC have been delineated into a main *Streptococcus gallolyticus* branch comprised of *S*. *gallolyticus* subsp. *gallolyticus (Sgg)*, *S*. *gallolyticus* subsp. *pasteurianus (Sgp)*, *S*. *gallolyticus* subsp. *macedonicus (Sgm*), the *Streptococcus infantarius* branch encompassing *S*. *infantarius* subsp. *infantarius (Sii)* and *S*. *lutetiensis* (intermediately called *S*. *infantarius subsp*. *coli*) and the single species *S*. *equinus* and *S*. *alactolyticus* [2, 3].

Members of the SBSEC are part of the gastrointestinal microbiota of humans and animals [1, 3, 4]. The fecal carriage rate of SBSEC in healthy adults ranges from 5% to 60% depending on isolation and identification techniques as well as geography [3, 5–7]. Members of the SBSEC are also described as opportunistic pathogens associated with various disease in humans and animals [7–13]. However, the major interest of these bacteria in human pathology, besides disease associations, is their controversial relationship with malignancies, mainly colorectal neoplasm [7, 12, 14–18]. Frequency of colorectal neoplasm varies widely from 6 to 67% among patients with SBSEC-related diseases. screening parameters, diagnostic tools and SBSEC species and subspecies seem to have a large impact on this widely varying frequency [7]. *Sgg* has been described as more strongly associated with colorectal cancer (CRC) and infective endocarditis in contrast to *Sgp* and *S*. *infantarius* branch members. The later two seem more frequently involved in hepatobiliary disorders and non-colonic cancer [7, 11, 14, 17, 19, 20]. Nevertheless, a colonoscopy is recommended for CRC screening in any patient with a SBSEC-related disease such as infective endocarditis, independent of the involved species. This was mainly attributed to the potential association of all SBSEC members in malignancy development [18, 21, 22]. Data supporting this association as well as documented infective endocarditis cases are however minimal for the *S*. *infantarius* branch and its two subspecies *Sii* and *S*. *lutetiensis* [7, 18, 23]. In addition, various *Sii* strains have been isolated from food products, mainly fermented dairy products (FDPs). *Sii* has been identified as predominant lactic acid bacteria (LAB) at 10^8^ bacteria per milliliter in spontaneously FDPs in various countries of sub-Saharan Africa, including in Côte d’Ivoire where 30–40% of FDPs are affected [24–28].

Comparative genomics of a *Sii* dairy isolate and the human-derived type strain revealed adaptation of the dairy isolate to the dairy environment similar to that of *Streptococcus thermophilus* [29]. However, the pathogenic background of *Sii* and SBSEC requires a comparative approach of multiple *Sii* isolates of various sources including humans for a comprehensive risk assessment. Human-derived *Sii* isolates, particularly those of Africa origin, and corresponding prevalence data are so far limited to a single East-African hospital-based study [6]. In West Africa, epidemiological data on human *Sii* isolates are lacking and knowledge is limited to dairy strains originating from Côte d’Ivoire and Mali [26, 28].

Population structure analysis by multi locus sequence typing (MLST) of *Sii* isolates indicated a division into three major clades comprised of (i) human blood isolates, (ii) East African dairy (EAD) isolates (iii) combination of West African dairy (WAD) and East African human (EAH) isolates [30]. This therefore suggested genetic differences between EAD and EAH isolates. This therefore implies the difficulty to portrait disease associations of human isolates, e.g. CRC and hemorrhoids, to those of dairy lineages in East Africa [6, 30]. However, the shared WAD EAH *Sii* clade, the current inexistence of a West African dairy lineage and the close relationship of some West African *Sii* isolates to those isolated from human bacteremia cases warrants a better East-West comparison and an enhanced health risk assessment in West Africa [30]. Therefore, we aimed in this study to identify risk factors for *Sii* fecal carriage corresponding with consumption of local FDPs. Thus, we hypothesized that the fecal carriage of *Sii* is higher in consumers of local FDPs. Given the existing *Sii* data for Mali and Côte d’Ivoire [26, 28], the dairy-producing area of Korhogo in Northern Côte d’Ivoire, close to the Malian border, would provide a suitable study area.

## Materials and methods

### Study site and population

**Fig 1:**
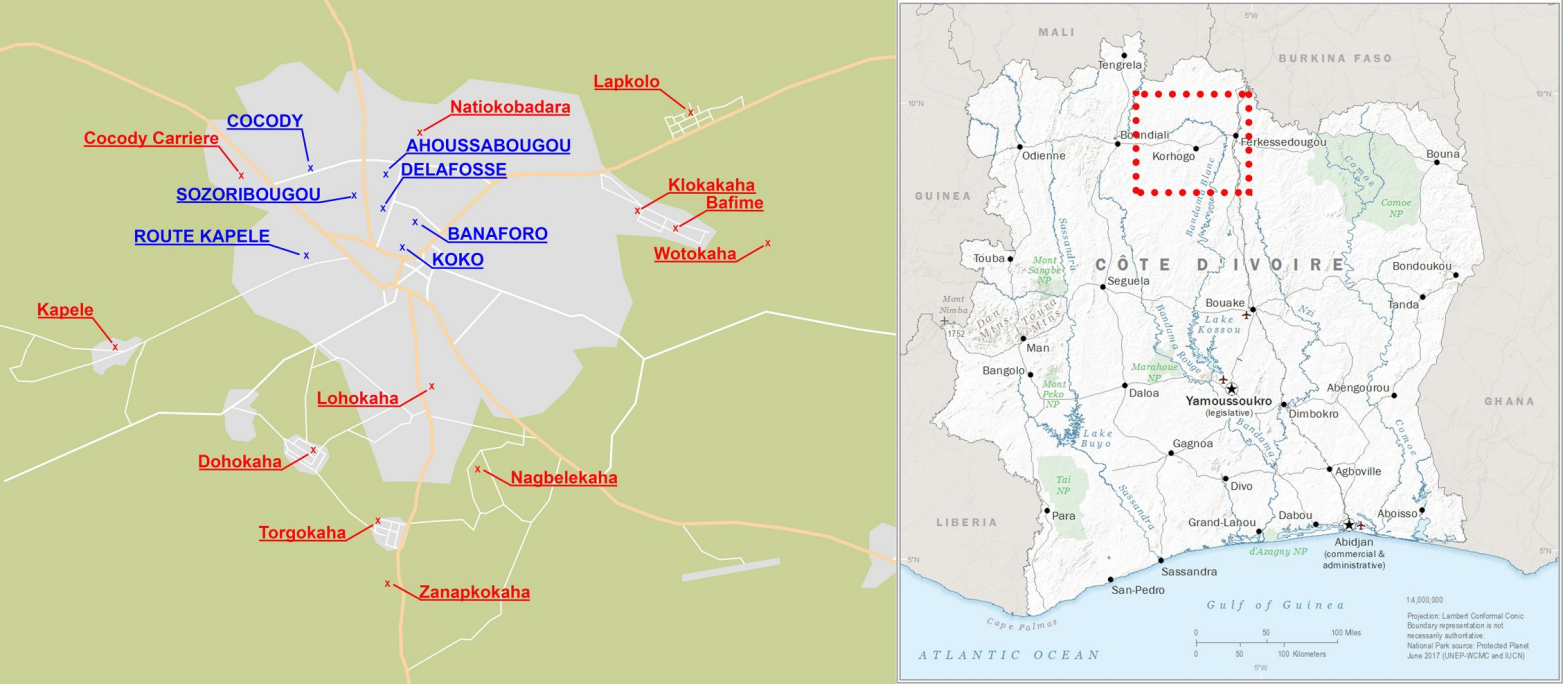
Illustration of the study site in the city of Korhogo and surrounding farms, northern Côte d’Ivoire. The illustrated map shows the selected neighborhoods (in blue capital font) and villages (in red) where the farms are located. Overview map of Côte d’Ivoire reprinted from the World Factbook.

A population-based cross-sectional survey was conducted between May and October 2014 in Korhogo, an agro-pastoral city in northern Côte d’Ivoire enrolling 595 households and 394 eligible participants. Before starting the main survey, preliminary interviews were performed in January 2014 with the head of the regional veterinary laboratory (LANADA), a representative of the Fulani community and milk sellers at the main market of the city. This allowed identifying the main local dairy products sold and the areas of high consumption. Seven neighborhoods were identified and selected for the present study: Cocody, Delafosse, Sozoribougou, Koko, Route Kapele, Banaforo and Ahoussabougou. In addition to these main sites, 31 small-scale dairy farms located in 10 hamlets, in a radius of 2 to 6 km of the city were also included for a possible comparison between outcomes from these areas and those from neighborhoods (Fig 1). A pilot survey was performed on a site outside those selected for the main study. In this pilot, the questionnaire was tested and stool samples were collected to improve procedures for data collection.

The study population for the survey consisted of adults aged 18 and older, consumers and non-consumers of dairy products permanently resident in the city. Besides these inclusion criteria, participants had to give their prior consent to participate in the interview and provide a fecal sample to be eligible for the study. Subjects who did not meet the inclusion criteria, as well as those who had undergone gastrointestinal surgery (<6 months before the survey) or who had received chemotherapy or radiotherapy and the pregnant women were not eligible.

### Field study sampling method of participants

#### Cluster design and field spotting

Numeric maps with boundaries of selected neighborhoods were designed using random Global Positioning System (GPS) coordinates registered on-site, the detailed numeric map of the city and Google Earth version 7.0. The number of participants to be sampled per neighborhood was allocated proportionally to the size of their population. Several random starting points were then generated around the on site GPS coordinates using a random point generator (http://www.geomidpoint.com/random/). Clusters for sampling were designed from starting points surrounded by four main streets. Furthermore, a specific location (school, mosque) was defined in each neighborhood as landmark from which tracks leading to the main starting points in a selected neighborhood were identified. The application “GPX viewer by vectura” was used to run the sampling tracks. Once a starting point was accurately spotted, the boundaries of the corresponding cluster were identified on site.

#### Participants sampling procedure

A multi-stage cluster sampling was conducted for participant’s selection. After selecting the clusters, a random selection of households was performed to be sampled along the boundaries of the main clusters in each block. The number of participants to be recruited per main starting point ranged from four to five depending on the population size and the number of clusters in each neighborhood. Thus, from the main starting house (which was not sampled), the next one anti-clockwise was sampled. Then, from the first household sampled, every third house was selected, until the number of interviews required was obtained or until the interviewer returned to the starting position. In cases where sampling of a household did not result in an interview (refusal case, non-eligible participants, empty house or non-inhabited place), the next household was directly selected and after that, every third house. The additional clusters were used when the required number of interviews was not reached after sampling of the main cluster in a block.

We assumed that one parcel or plot of land corresponded to one household. In cases where more than one household was found inside the same parcel, they were considered as a single household and one participant was randomly selected from all the residents if the parcel belonged to the same family. If different families resided in the same parcel, one out of the households was sampled randomly as following: from the entry of the plot of land, all households were numbered anti-clockwise. A number was selected on a single use random digit table (sequence of digits from 0 to 9, randomly arranged in a table). The household with the corresponding number was chosen for the survey. The number used on the random digit table was excluded from further use.

Afterwards, participant selection in each household was conducted. Only one participant was randomly selected per household according to the following procedure: the members of the household present at home at the time of the survey and meeting the eligibility criteria were ranked and numbered from the youngest to the oldest. Using as well a random digit table, a number was selected and the person with the corresponding number was selected for the survey.

### Data Collection

#### Questionnaire survey and anthropometric measurements

A structured questionnaire, based on that of Kenyan hospital-based study [6], was adapted to local customs, diet and habits. The questionnaire was used to identify potential factors associated with the fecal carriage of *Sii*. It covered information on demographic, socio-economic and behavioral determinants. This included smoking status, alcohol consumption, and physical activity as well as nutritional habits and the relation to livestock in daily live and work.

Assessed food items included mainly dairy products and food groups such as meats (red meat and poultry), fish, eggs, pulse, tea, coffee, fruits, vegetables, tubers, plantain, cereals and dietary supplements. Participants were asked for their usual food consumption frequency per food item. Frequencies were split into five categories: 1–2 days per month; 1–2 days per week; 3–5 days per week; 6–7 days per week and unknown frequency. For each food item, the average quantity of consumption was recorded. The portion size was estimated using household units (teaspoon, cup 1) and metric units (milliliter, kilogram).

Information on participants' health status were collected. This included reporting of diseases diagnosed over the past year by a health professional as well as self-reported clinical symptoms that they think they have suffered from during the 5–7 days preceding the survey. The questionnaire was administered in French but also in “*Dioula*”, a local West African language and "*Senoufo*", the local dialect. The height and weight of each participant were measured by using standard procedures [31].

#### Stool and milk sample collection

Samples included stool from each participant and animal-derived milk from each household if available. For stool collection, a sterile screw-capped tube (Sarstedt, Sevelen, Switzerland) with integrated spoon was given to each participant after the interview. The the procedure for self-collection of a stool sample was thereby explained. The specimens were packed in stomacher bags and transported in cool boxes with ice packs to the regional laboratory. Fresh and fermented milk samples were also collected in households where available. Fermented milk samples were further differentiated into traditional FDP (tFDP) from the informal or household sector and industrial FDP. Milk samples were taken using sterile screw-capped tubes and transported in cool boxes with ice packs separated from those boxes used for stool samples. Upon arrival at the laboratory the same day, all samples were stored at -20°C until subsequent *Sii* isolation as previously done [24, 28]. This procedure is enabled by the ability of the targeted SBSEC bacteria, belonging to fecal streptococci, to survive several freeze-thaw cycles and the protective effects of carbohydrates, fat and proteins in milk and feces [32, 33]

### Microbiological and molecular laboratory methods

#### Enumeration and selective isolation of SBSEC members from fecal and milk samples

Mitis-salivarius (MS) agar medium with tellurite solution 1% (Becton Dickinson, Allschwil, Switzerland) was used for enumeration and isolation of presumptive SBSEC and *Sii* from fecal samples (n = 385) as previously described [6]. Briefly: after thawing, an aliquot (about 100 mg) of each fecal sample was homogenized in 9 mL of sterile peptone saline solution (1% peptone, 0.85% NaCl) using a vortex. The suspension was serially diluted and 100 μL plated onto MS agar followed by incubation for 48 hours aerobically at 37°C. Out of the five plates seeded, three with desired black or blue colony types in pinpoint or slimy appearance and cfu counts <300 were selected for bacteria enumeration and isolation. One to three single colonies were picked per morphology type and streak purified.

The enumeration and isolation of presumptive members of the SBSEC from milk products (n = 52) was performed using M17 agar media (Biolife, Milan, Italy) as previously described [24]. The isolation procedure using MS and M17 agar media therefore matched that of previous human stool and dairy product sampling, respectively, to ensure continued comparability of data [6, 24, 28].

#### Procedures for bacterial identification

Preliminary identification was performed via assessment of the catalase activity of purified strains using hydrogen peroxide (3% H_2_O_2_) [20]. Only catalase-negative isolates were further processed. DNA extraction for molecular biology tests was performed on purified single colonies using a short cell lysis in a Tris-EDTA triton-X-based buffer [34]. Identification of presumptive SBSEC isolates was further carried out through SBSEC-specific PCR assays based on sequence analysis of 16S rRNA (primer set 16S-SBSEC-fw, 16S-SBSEC-3-fw, 16S-SBSEC-4-fw, 16S-SBSEC-5-fw and 16S-inf-rev) and *groEL* (primer set *groEL*-fw and *groEL*-rev) genes as previously described [28, 35, 36].

#### Determination of population structure of SBSEC members

The population structure of SBSEC isolates was analyzed via the SBSEC MLST assay and deposited on pubmlst.org as previously described [35]. Clonal complex (CC) calculations were performed in Phyloviz 2.0 and eBURSTv3 [37, 38] for groups of sequence types (STs) sharing 7 or more out of 10 alleles with at least one other member of this group [35]. The complete set of strains including reference strains used for population structure calculations is described in S1 Table.

### Statistical analysis

The questionnaire data were double-entered in Microsoft access 2010 and compared with EpiInfo version 3.5.3 (Center for Diseases Control and Prevention; Atlanta, United States of America). Data were analyzed using STATA version 12.1 (Stata Corporation, College Station, Texas 77845 United States of America). The household wealth index to assess participants' socioeconomic status was created using the principal components analysis (PCA) of 13 household assets as previously described [39]. To determine the risk factors for *Sii* fecal carriage, logistic regression was performed. Variables with p ≤ 0.2 and those, which are biologically plausible, were selected for the multivariate regression model. The selected explanatory variables were then tested for multicollinearity and fitted into the final multivariable logistic regression model.

### Ethical consideration

The study was approved by the national ethics committees of Côte d'Ivoire (N 018/MSLS/CNER-dkn), Eidgenössische Technische Hochschule (ETH) Zurich, Switzerland (EK2013-N-78) and Kantonale-Ethik-Kommission Zurich, Switzerland (StV-Nr47/14). The study was conducted in accordance with the Declaration of Helsinki [40]. Informed written consent was obtained from all participants prior to enrollment in this study.

## Results

### Study population characteristics

**Fig 2:**
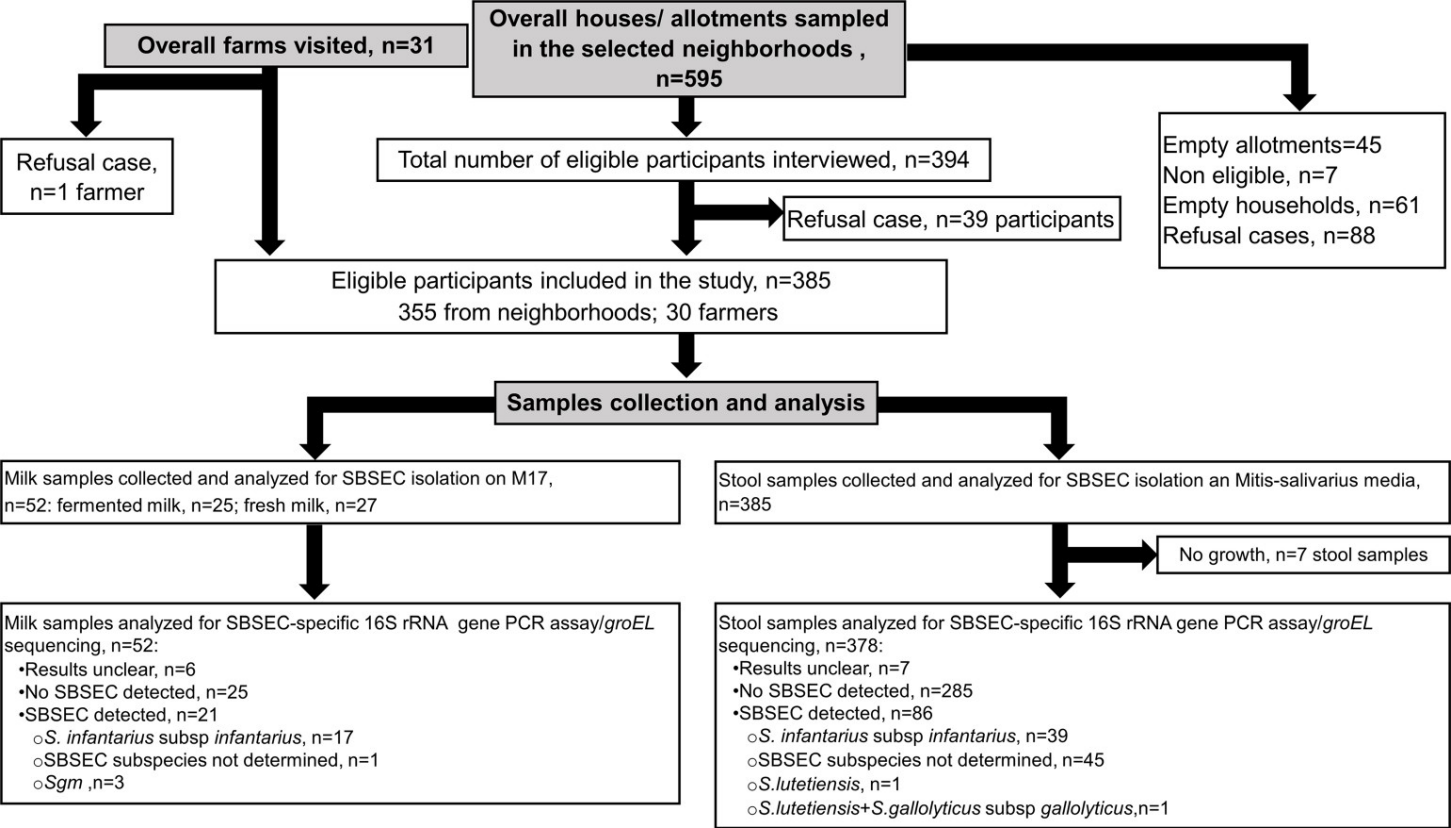
Data flow diagram of participant selection procedure and SBSEC/*Sii* detection from stool and milk sample analysis.

Out of the 355 participants initially interviewed in the neighborhoods, 39 failed to provide a stool sample (Fig 2). These participants were excluded and replaced. Overall, 394 subjects were randomly selected in the seven neighborhoods within 163 clusters. After curation of participants using the defined inclusion and exclusion criteria, a total of 385 participants (355 from neighborhoods and 30 farmers) were included in the analyses for basic demographic, economic and health characteristics of the study population (Table 1). The study population was young (median age of 35 years (inter quartile range = 27–48)), with 62.8% (242/385) females. More than one third of participants were overweight with a prevalence of 37.7%. Ulcer and hypertension were the second more frequent morbidities observed, with prevalence of 21.8% and 15.2%, respectively.

**Table 1:** Demographic, economic, behavioral and health characteristics of the study population.

Variables	Description	% (n)^a^
Age (Years)	18–30	36.3 (137)
	31–60	54.1 (204)
	>60	9.1 (36)
Gender	Male	37.1 (143)
	Female	62.9 (242)
Birth country	Côte d'Ivoire	87.3 (336)
	Other^b^	12.7 (49)
Level of education	Ever attended school	47.3 (182)
	Koranic school	17.7 (68)
	Conventional school	35 (135)
	Primary	37 (50)
	Secondary	54.8 (74)
	University	8.2 (11)
Number of person per house	Single person	3.7 (14)
	2–4	19.5 (74)
	5–10	40.1 (152)
	>10	36.7 (139)
Water supply of the house^c^	Tap water	41.4 (157)
	Protected well	63.6 (241)
	Water hole	1.8 (7)
	Pump	4.7 (18)
Socioeconomic status	Lowest quartile	26.2 (101)
	Second quartile	23.9 (92)
	Third quartile	25.2 (97)
	Highest quartile	24.7 (95)
Lifestyle		
Dietary habits	Regime without milk products	3.2 (11)
	Regime without pork	84.8 (324)
Tobacco consumption	Never smoked	82 (314)
	Current consumers	12.3 (47)
	Past-consumers	5.7 (22)
Alcohol consumption	Non-consumers	89.2 (339)
	Current consumers	7.9 (30)
	Past-consumers	2.9 (11)
Health characteristics		
BMI^d^	Underweight, BMI < 18.5	9.6 (35)
	Normal, 18.5 ≤BMI ≥ 24.9	52.6 (191)
	Overweight ≥25	37.7 (137)
Morbidities^c^	Heart disorders	4.2 (16)
	Hypertension	15.2 (58)
	Diabetes	2.4 (9)
	Arthritis	2.4 (9)
	Ulcer	21.8 (83)
	Lung problems	1.8 (7)

^a^Numbers might not add up to 385 because of missing values

^b^Burkina Faso, Mali, Niger, Ghana, Togo

^c^multiple answers possible

^d^BMI: body mass index, (Weight (Kg) / Height (m^2^)).

### Consumption patterns of local milk products

**Fig 3:**
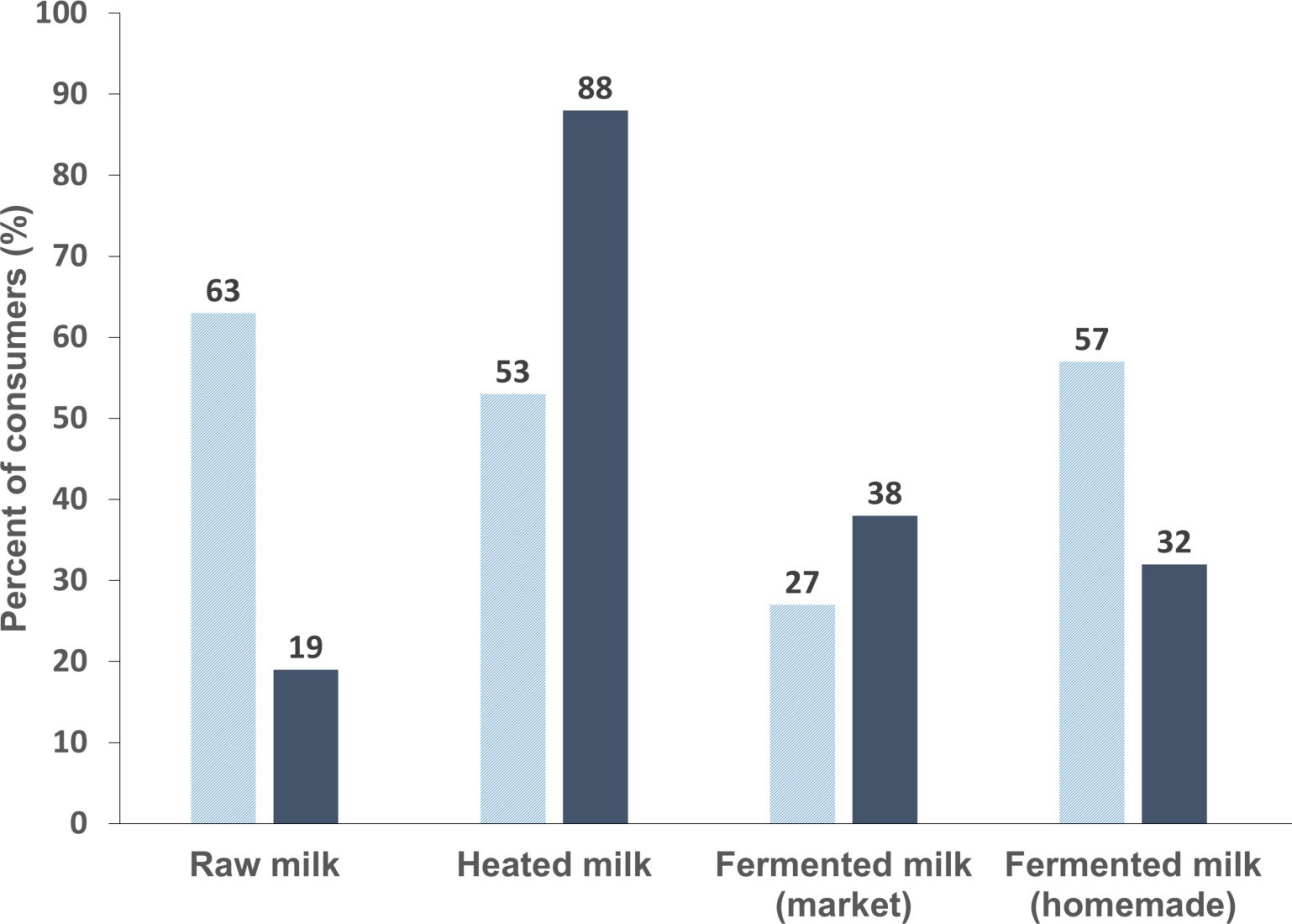
Proportion of different local dairy products consumed in farms (light colored columns) in comparison to urban households (dark columns).

Overall, 79.7% of the participants were regular consumers of local dairy products, which were produced exclusively from local cow milk. Most of consumers (84.7%) stated that they heat their milk prior to consumption, while 23.8% asserted that they consume milk directly after milking (farmers) or purchase (households). Regarding FDPs bought at market and homemade products, the proportion of consumers were 37.1% and 34.8%, respectively. Stratified by farm and urban households, significantly more people in farms consumed raw milk (63% vs 19%, p < 0.0001) and homemade FDPs (57% vs 32%, p = 0.008) compared with households in urban areas, where more people heated milk (88% vs 53%, p < 0.0001) before the consumption (Fig 3).

### *Sii* prevalence and determinants

#### *Sii* fecal carriage

The overall prevalence of SBSEC and *Sii* was 23. 2% (CI 95% = 18.9–27.5) and 12.0% (CI 95% = 8.4–15.5), respectively. Prevalence of *Sii* was significantly higher among subjects over 60 years of age and those born in Sahelian countries (Table 2). The fecal carriage rate of *Sii* was slightly higher in males and in the lowest socioeconomic quartile but the relationships were not statistically significant. Concerning the consumption of local dairy products, the overall fecal carriage rate of *Sii* in consumers of these foods was similar to that of non-consumers (12.1% vs 11.6%; OR: 1.0; 95% CI: 0.5–2.4; p = 0.92). Moreover, prevalence of *Sii* was higher in consumer of raw milk (19.0%) and homemade FDPs (16.3%) but the observed differences were not statistically different. However, the *Sii* fecal carriage rate was significantly higher in consumers of artisanal butter compared with non-consumers (57.1% vs 10.1%, OR: 11.9, 95% CI: 3.9–36.6).

**Table 2:** Univariate analysis of risk factors for *Sii* fecal carriage among the study population.

Variables	Category	%(n positive/ n total)	OR^a^	95% CI^b^	p-value
Gender	Female	10.3 (21/204)	1.00		
	Male	14.7 (18/122)	1.5	0.8–2.9	0.23
Age (Years)	18–30	10.3 (12/117)	1.00		
	31–60	10.5 (18/171)	1.0	0.5–2.2	0.94
	>60	24.2 (8/33)	2.8	1.0–7.6	0.04
Birth country	Other country	10.6 (31/291)	1.00		
	Sahelian country	22.9 (8/35)	2.5	1.0–5.9	0.04
Residence area	Neighborhoods	9.9 (30/303)	1.00		
	Farms	39.1 (9/23)	5.8	2.3–14.6	<0.001
Socioeconomic status	Lowest quartile	16.3 (14/86)	1.00		
	Second quartile	8.1 (6/74)	0.4	0.2–1.2	0.13
	Third quartile	15.5 (13.84)	0.9	0.4–2.1	0.9
	Highest quartile	7.3 (6/82)	0.4	0.1–1.1	0.08
Livestock ownership	No	10.4 (26/251)	1.00		
	Yes	20 (12/60)	2.2	1.0–4.6	0.04
Contact with livestock					
Livestock nearby dwelling place	No	10.9 (21/192)	1.00		
	Yes	14.7 (17/116)	1.4	0.7–2.8	0.34
Daily routine with livestock	No	10.4 (29/279)	1.00		
	Yes	31 (9/29)	3.9	1.6–9.3	0.002
Daily routine with livestock primary product	No	10.2 (29/285)	1.00		
	Yes	39.1 (9/23)	5.7	2.3–14.3	<0.001
Only some people at home in contact with livestock	No	12.3 (36/292)	1.00		
	Yes	12.5 (2/16)	1.01	0.2–4.6	0.984
No contact with livestock	No	14.4 (21/146)	1.00		
	Yes	10.5 (17/162)	0.7	0.4–1.4	0.302
Type of dairy products consumed					
Raw milk	No	10 (20/199)	1.00		
	Yes	19.0 (11/58)	2.1	0.9–4.7	0.07
Heated milk	No	20 (8/40)	1.00		
	Yes	10.6 (23/217)	0.5	0.2–1.1	0.09
FDP from market	No	14.5 (24/165)	1.00		
	Yes	7.6 (7.92)	0.5	0.2–1.2	0.12
Homemade FDP	No	9.9 (17/171)	1.00		
	Yes	16.3 (14/86)	1.8	0.8–3.8	0.14
Artisanal butter	No	10.1 (31/308)	1.00		
	Yes	57.1 (8/14)	12	3.9–36.6	<0.001
Industrial butter	No	12 .6 (27/215)	1.00		
	Yes	11 (12/109)	0.9	0.4–1.8	0.7
Industrial cheese	No	12.3 (39/317)	1.00		
	Yes	0 (0/5)	1		
Ice cream	No	12.5 (36/289)	1.00		
	Yes	3.1 (1/32)	0.2	0.03–1.7	0.15
Milk powder	No	13.2 (20/151)	1.00		
	Yes	10.9 (19/174)	0.8	0.4–1.6	0.52
Imported FDP	No	13.4 (39/291)	1.00		
	Yes	0 (0/25)	1		
Yogurt	No	14.3 (32/223)	1.00		
	Yes	6.9 (7/102)	0.4	0.2–1.0	0.06
Other foods					
Eggs	No	12.5 (8/64)	1.00		
	Yes	11.5 (30/260)	0.9	0.4–2.1	0.83
Poultry with skin	No	8.9 (12/135)	1.00		
	Yes	13.8 (26/188)	1.6	0.8–3.4	0.18
Poultry without skin	No	11.9 (33/278)	1.00		
	Yes	11.1 (5/45)	0.9	0.3–2.5	0.88
Can meat	No	12.2 (37/303)	1.00		
	Yes	5 (1/20)	0.4	0.1–2.9	0.35
Can fish	No	11.9 (28/236)	1.00		
	Yes	11.6 (10/86)	0.97	0.4–2.1	0.95
Pig meat	No	11.5 (34/296)	1.00		
	Yes	16 (4/25)	1.5	0.5–4.5	0.51
Red meat	No	14.2 (18/127)	1.00		
	Yes	10.1 (19/189)	0.7	0.3–1.3	0.27
"Choukouya" (Braised meat)	No	11.4 (15/132)	1.00		
	Yes	12 (23/191)	1.1	0.5–2.1	0.85
Fish	No	25 (2/8)	1.00		
	Yes	11.5 (36/314)	0.4	0.1–2	0.26
Seafood	No	12.2 (34/279)	1.00		
	Yes	2.7 (1/37)	0.2	0.03–1.5	0.12

^a^OR: odds ratio

^b^CI: confidence interval.

Regarding residence area, more *Sii* fecal carriers were found among participants from farms compared with urban households (39.1% vs 9.9%; OR = 5.8; 95% CI: 2.3–14.6). Similarly, there were significantly more fecal carriers of *Sii* among livestock owners (OR = 2.2; 95% CI = 1.02–4.6), in subjects with daily routine with livestock (OR = 3.9; 95% CI = 1.6–9.3) and those with daily routine with livestock primary products (OR = 5.7; 95% CI = 2.3–14.3).

In the multivariate logistic regression analysis (Table 3), the direct livestock contact was strongly associated with *Sii* fecal carriage (OR = 6.2; 95% CI = 1.03–37.5). Subjects over 60 years of age had a higher risk for *Sii* carriage (OR = 1.6; 95% CI = 0.4–6.1), but this association was not statistically significant. Similarly, consumers of unboiled milk (OR = 1.5; 95% CI = 0.4–5), homemade FDPs (OR = 1.6; 95% CI = 0.6–4.1) and poultry with skin (OR = 2.1; 95% CI = 0.8–5.7) showed a higher risk for *Sii* fecal carriage without statistically significant differences observed. These overall calculations were derived from the analysis of 4450 isolates of fecal origin (S3 Table).

**Table 3:** Multivariate logistic regression analysis of risk factors for *Sii* fecal carriage.

Variables	Category	%(n positive/ n total)	OR^a^	95% CI^b^	p-value
Gender	Female	10.3 (21/204)	1.00		
	Male	14.7 (18/122)	0.8	0.3–2.6	0.75
Age (Years)	18–30	10.3 (12/117)	1.00		
	31–60	10.5 (18/171)	0.9	0.3–2.4	0.84
	>60	24.2 (8/33)	1.6	0.4–6.1	0.49
Birth country	Other country	10.6 (31/291)	1.00		
	Sahelian country	22.9 (8/35)	0.3	.1–2	0.24
Socioeconomic status	Lowest quartile	16.3 (14/86)	1.00		
	Second quartile	8.1 (6/74)	0.4	0.1–1.7	0.24
	Third quartile	15.5 (13.84)	1.2	0.4–4.3	0.72
	Highest quartile	7.3 (6/82)	0.6	0.1–2.6	0.46
Direct livestock contact^c^	No	10.4 (29/278)	1.00		
	Yes	30 (9/30)	6.2	1.04–37.5	0.04
Raw milk	No	10 (20/199)	1.00		
	Yes	19.0 (11/58)	1.5	0.4–5	0.54
Heated milk	No	20 (8/40)	1.00		
	Yes	10.6 (23/217)	1.01	0.2–4.2	0.98
FDP from market	No	14.5 (24/165)	1.00		
	Yes	7.6 (7.92)	0.6	0.2–1.7	0.32
Homemade FDP	No	9.9 (17/171)	1.00		
	Yes	16.3 (14/86)	1.6	0.6–4.1	0.29
Yogurt	No	14.3 (32/223)	1.00		
	Yes	6.9 (7/102)	0.4	0.1–1.3	0.13
Poultry with skin	No	8.9 (12/135)	1.00		
	Yes	13.8 (26/188)	2.1	0.8–5.7	0.14
Red meat	No	14.2 (18/127)	1.00		
	Yes	10.1 (19/189)	0.6	0.2–1.5	0.29

^a^OR: odds ratio

^b^CI: confidence interval

^c^Daily routine with livestock and/or livestock primary products. Variables with a level of p 0.2 at the univariate analysis and those, which are biologically plausible, were selected for the model. However, those among them with very low observation in have not been fitted into the model (industrial cheese, ice cream, imported fermented dairy products (FDP), seafood). Correlation test has revealed strong association between such explanatory variables: residence area (A), livestock ownership (B), daily routine with livestock and/or livestock primary product (C) and artisanal butter (D). A: B(r = 0.6), A: C (0.9); A: D (0.8); B: C (0.6); C: D (0.7). The explanatory variable "B" was selected for the model.

#### Milk samples

*Sii* strains were isolated from 52 milk samples, with an overall prevalence of 37.8% (CI 95% = 23.0–52.5). Prevalence of *Sii* in fermented and raw milk samples was 45.4% and 30.4% respectively. These rates were not statistically different. These overall calculations were derived from the analysis of 79 and 84 isolates of fermented and raw milk origin, respectively (S3 Table).

### SBSEC population structure

**Fig 4:**
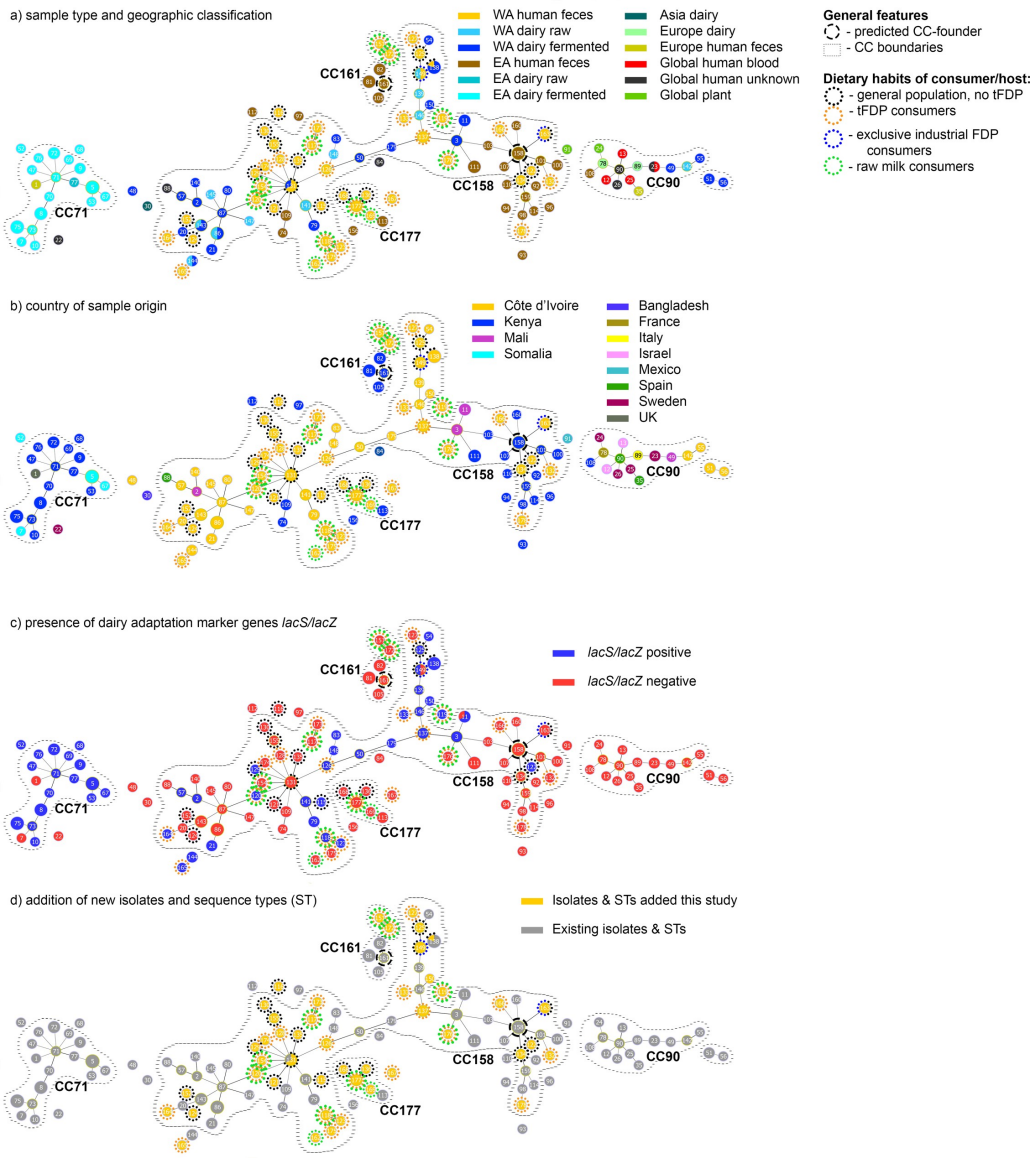
Phylogenetic tree of *Sii* of dairy, plant and human origin based on MLST profiles. *Sii* isolates and meta data are indicated concerning sample type and geographic classification (A), country of sample/isolate origin (B) and presence of *lacS/lacZ* dairy adaptation (C) and highlighting of new isolates and STs added in this study (D).

A total of 53 SBSEC isolates collected during this study were processed by MLST. This included 50 *Sii*, one *Sgg*, one *Sgp* and one S. *lutetiensis*. In addition, MLST data was extracted from 37 SBSEC genomes available on GenBank to optimize the general SBSEC portfolio for comparisons. In combination with SBSEC STs derived from a parallel conducted farm-level study in the Korhogo area (Sanhoun et al., submitted for peer-review) and already deposited STs, this yielded a total of 271 SBSEC isolates in the current database generating 210 unique STs. The majority of isolates were identified as *Sii* and clustered in major CCs CC158, CC90, CC71, CC177 and CC161. This was in addition to a few two-ST complexes and multiple singletons (Fig 4 and S1 Fig).

The majority of new isolates joined CC158 to yield a total of 96 isolates representing 68 STs. CC158 is comprised of a mix of Ivorian and Mali dairy isolates as well as Ivorian and Kenyan human isolates. The predicted founder ST158 is of Kenyan human origin. Further key nodes in CC158 are represented by Ivorian-derived ST137, ST131 and ST87 featuring a mix of dairy and human fecal sources. Particularly ST87 represents a key node for the majority of WAD *Sii* isolates (Fig 4). It is comprised of dairy isolates and STs originally described from the Abidjan area of Côte d’Ivoire and Mali as well as four novel STs and isolates obtained during this study. Dairy adaptation marker genes *lacS* and *lacZ* were detected in 5/15 STs connected to node ST87.

The connection between ST87 and ST158 via ST131 and ST137 as main intermittent nodes was facilitated by a series of STs representing Ivorian human-derived and Malian dairy-derived, dairy-adapted *Sii* isolates. The direct connection was comprised of four human-derived STs (ST120, ST126, ST137 and ST103), three dairy-derived STs (ST50, ST179 and ST3) and one ST shared between human and dairy sources. All dairy-derived STs also featured *lacS/lacZ* dairy adaptations with the exception of ST131-isolates. Out of the human-derived STs (ST120, ST126, ST137 and ST103), *lacS/lacZ* dairy adaptations were found in ST120, ST126, and ST137. The isolates yielding these STs were obtained from tFDP consumers of which one also consumed raw milk. ST137 furthermore represents a main node for a side branch for STs representing multiple dairy-adapted *Sii* STs (9/10 STs including ST137) derived from West African human and dairy sources. Among the six human-derived STs (ST125, ST127, ST133, ST137, ST138 and ST149), five also featured *lacS/lacZ* of which two STs were derived from tFDP consumers. The remaining three (ST125, ST138 and ST149) were obtained from participants not consuming tFDP or raw milk. Similarly, out of the total of 15 STs representing *lacS/lacZ* dairy-adapted variants obtained from human participants, nine STs were linked to tFDP consumers. This suggeststhe potential for transfer of *Sii* via tFDP and a possible reservoir of *Sii* in tFDP.

A total of 40 isolates were obtained as pairs originating either from human and dairy samples of the same household (n = 14), raw and FDPs from the same household (n = 6) or from the same human participant (n = 20) (S2 Table). Among dairy product isolates of participant AR007, two out of six isolates and STs (ST138/AR007A22.1 and ST139/AR007B22.1) featured closely related STs and shared dairy adaptation *lacS/lacZ* (S2 Table). In this study, closely related STs and isolates such as ST143/AR004A26.1 and ST152/AR004F6.1, both non-dairy-adapted, were isolated from dairy and fecal samples from participant AR004, respectively. In addition, the same dairy sample of participant AR004 comprised a dairy-adapted isolate ST138/AR004A26.3. This isolate was not obtained from fecal samples and only featured a distant relationship within CC158 to ST143 and ST152. Similarly, samples of participant AR022 featured dairy-adapted isolates of ST179 in FDPs. However, fecal samples returned only non-dairy-adapted isolates of ST172. Participant AR038 returned non-dairy-adapted isolates of ST86 and ST143 from dairy products whereas the fecal sample returned a distant non-dairy-adapted isolate of ST154. This suggests that a link between the two habitats may exist in some cases only.

In contrast, participant AR037 returned dairy-adapted ST138 and ST119 from dairy and fecal samples alike. Parallel existence of dairy-adapted and non-adapted STs in fecal samples of participants AR017, CL026, GKDT016, TKF022, TKF029 and dairy samples of participants AR004, AR016 and AR064 (S1 Table) suggests bidirectional exchange of strains between the dairy and the human habitat.

No new connections via MLST were observed for the Ivorian *Sgp* (ST153) *S*. *lutetiensis* (ST171) and *Sgg* (ST170) despite the addition of 37 GenBank-derived strains for *Sgg* (n = 13), *Sgp* (n = 1), *S*. *lutetiensis* (n = 3) and *S*. *equinus* (n = 20) (S1 Fig). Only for *Sgg*, the formation of a CC comprised of ST16, ST32, ST183 and ST205 of European infective endocarditis isolates as well as a CC comprised only of ST203 and ST204 of African primate nasal swabs was observed (S1 Fig).

## Discussion

This study aimed to fill the knowledge gaps regarding the epidemiology of *Sii* in West African regions. For this purpose, the prevalence and risk factors for *Sii* carriage were investigated, as well as the relationship of *Sii* strains of West and East African origin.

The SBSEC prevalence of 23.2% in our study resulted from a population-based cross-sectional survey. To the best of our knowledge, this is the first evaluation of SBSEC fecal carriage in the general population. However, the actual SBSEC prevalence might have been underestimated given that our assay was focused at isolating *Sii*. *Sii* is the predominant subspecies isolated in the present study, with a true prevalence of 12.0%. Nevertheless, similar SBSEC and *Sii* fecal carriage rates were observed in prior surveys [6, 41]. Particularly, the SBSEC prevalence is similar to that of the Kenyan hospital-based survey, which used a similar isolation and identification approach [6]. It might thus provide good justification for an East-West comparison of SBSEC and *Sii* prevalence in humans to be related to a number of local factors, notably nutritional and environmental factors.

A relationship between the consumption of dairy products and *Sii* carriage was observed in this study. Indeed, *Sii* was significantly more prevalent in consumers of artisanal butter than in non-consumers. This food also called “naré” in Fulani language, is a dairy product obtained from milk fat (raw or boiled milk) after fermentation and using traditional extraction processes. In contrast, this association was not observed with the other local dairy products. The variation noted in the relation of dairy products with *Sii* carriage lead us to imagine the possibility that the fermentation level or process could influence this relationship.

In addition, MLST phylogeny suggested a link of *Sii* human isolates with dairy products. This was suggested by the presence of the dairy adaptation marker genes in some *Sii* isolates obtained from tFDPs consumers. This was not the case for EAH *Sii* isolates [30]. More interestingly, although in small numbers, *Sii* human-dairy product strain pairs were also observed. This might suggest the contribution of tFDPs to the gut colonization by *Sii*, especially since *Sii* dairy strains may survive in the gastrointestinal tract. This finding in combination with the unexpected high proportion of *Sii* carriers in participants from Sahelian countries hints toward a potential contribution of cultural determinants, dietary habits and livestock culture to the gut colonization by these microorganisms. This should warrant investigations also in comparison to European or Asian or North American countries where comparable data is missing.

Interestingly, proximity to livestock seems to be a risk factor for *Sii* carriage. An analysis of the participants' relation to animals revealed a significantly higher proportion of *Sii* carriers only among those in direct contact with livestock and livestock primary products. Importantly, this closer livestock contact was identified as a risk factor in the multivariate analysis suggesting the possible transmission of *Sii* from animals to humans. This supports previous assumptions of hospital-based surveys, which ascribed the high frequency of SBSEC-related infections among subjects living in rural areas to the closer contact with animals [42]. Similarly, a recent study involving *Sgg* identified the closer animal contact as well as usage of manure as risk factors for *Sgg* transmission to humans [5]. In addition, *Sgg* was described as potential zoonotic bacteria due to shared STs between cattle and humans isolates [5]. Epidemiological data regarding *Sii* from animal source are lacking. However, selected lineages of *Sii* strains were involved in human infections and thus, the possibility of zoonosis should be investigated also for *Sii*.

The high rate of *Sii* fecal carriers observed in elderly is consistent with previous reports noting an association between advanced age and occurrence of SBSEC-related infection [21, 43–45]. This fact seems to be related to the weakening of the body's immune defenses and frequency of morbidities in this age group [44]. This therefore indicates that SBSEC members could act as opportunistic pathogens.

*Sii* strains have been isolated from FDPs with a prevalence similar to that previously reported in Côte d'Ivoire [28]. Unexpectedly, *Sii* strains have also been isolated from fresh milk. This adds a potential new reservoir to FDPs as the so far exclusive *Sii* reservoir in the dairy niche [28]. It remains to be investigated whether cross-contamination between FDPs and raw milk through the environment, workers or utensils was responsible for this finding. This highlights once again the interest for in-depth investigations on the ecology and potential reservoirs as well as pathways of contamination by *Sii* at the human-animal-environment interface.

Population structure analysis via MLST revealed the close relationship of West and East African human *Sii* and WAD *Sii* through the formation of a main CC158. The current ST- and isolate set led to a predicted founder of CC158 as ST158 of Kenyan human origin. While the link between East and West African isolates is well-supported by the presented data, the founder prediction and thus the possible evolution of *Sii* in Africa remains to be confirmed by in-depth analysis of additional isolates. Nevertheless, the presence of dairy adaptations offers some suggestions for their evolution: the *Sii* isolates in CC158 displayed presence and absence of dairy adaptations alike, as observed via marker genes *lacS* and *lacZ*. Isolates with STs neighboring ST137 featured such dairy adaptations mainly in a small branch derived from ST141 (adjacent to ST131), a large branch derived from ST137 and the STs connecting ST131 with ST137. This allowed for a possible separation of CC158 into these predominantly dairy-adapted branches and two main human-derived branches. The two predominantly human-associated branches thereby concentrated around ST131 (for West Africa) and ST158 (for East Africa). Except for the ST158 branch, no branch seemed to be exclusive to either human or dairy origin. Among the dairy-adapted branches, dairy products or the isolation from consumers of dairy products was the predominant isolate background. In the other branches and isolates, dairy adaptation was less prevalent and the isolation origin was less frequently associated with dairy consumers. This was particularly observed in STs linked to ST87 and its non-dairy-adapted isolates originating from dairy sources. This suggests for one the divergence of mainly dairy-adapted lineages from likely human commensal lineages. Second, bidirectional transfer of dairy-adapted strains to humans via FDP consumption and non-dairy-adapted human commensal strains to dairy products is a possible explanation for the presence of multiple STs of adapted and non-adapted isolates in the same habitat. The observation of multiple separate branches featuring dairy-adapted STs and the alternation between dairy-adapted and non-dairy-adapted STs in several branches strongly supports earlier indications for an evolution driven mostly by the occurrence of multiple recombination events rather than a single ancestral lineage. These findings would therefore support further investigations to better define the ecology and reservoirs of *Sii* in Africa. This would allow to better define marker strains for a safety assessment of *Sii* in relation to consumption via food.

## Conclusion

This study provides the first data on SBSEC/*Sii* prevalence in the general population. Our findings allowed confirmation of earlier observations regarding determinants, especially demographic, environmental and nutritional factors that might influence the fecal carriage of SBSEC members. Such data was previously only obtained from hospital-based studies and was thus less representative for the general population. We noted a significantly higher rate of *Sii* fecal carriage in elderly, in consumers of artisanal butter, as well as in subjects in close contact with livestock and livestock primary products. The livestock proximity has been identified as a risk factor for *Sii* fecal carriage, indicating a possible transmission of *Sii* from animals to humans. The population structure of *Sii* in Côte d’Ivoire revealed a close relationship across human and dairy isolates in East and West Africa with a predicted founder linking back to a Kenyan human isolate. The presence of dairy adaptations and isolation origins however suggest the establishment of multiple dairy lineages and an evolution driven by recombination rather than clonal development. In-depth investigations on the ecology, potential reservoirs and pathways of contamination by *Sii* at the human-animal-environment interface in Africa will be needed in comparison to yet to be collected data from Europe, Asia and the Americas to further elucidate the various roles of *Sii*.

## Supporting information

S1 Fig Phylogenetic tree of SBSEC sequence types and isolates of dairy, plant and human origin. The tree is based on MLST profiles and colored according to SBSEC species with additional indications for CC founders and dietary habits of the host.
(TIF)

S1 Table SBSEC strains used in this study for population structure analysis. All 271 SBSEC strains analyzed via MLST including relevant metadata are listed.
(XLS)

S2 Table Key characteristics of the study participants featuring isolate pairs from fecal and dairy samples. Study participants featuring isolate pairs from fecal and dairy samples with participant’s dietary habits and corresponding isolate information on sequence type (ST) and dairy adaption marker genes *lacS/lacZ*.
(XLSX)

S3 Table Complete list of fecal and milk isolates of the study. All 4613 isolates processed in this study including identification results are listed.
(XLSX)
