# Supplementary figures and images for: Risk factors for the carriage of Streptococcus infantarius subspecies infantarius isolated from African fermented dairy products
Source: PLoS One. 2019 Nov 27;14(11):e0225452. doi: 10.1371/journal.pone.0225452 (PMC6881063; doi:10.1371/journal.pone.0225452)

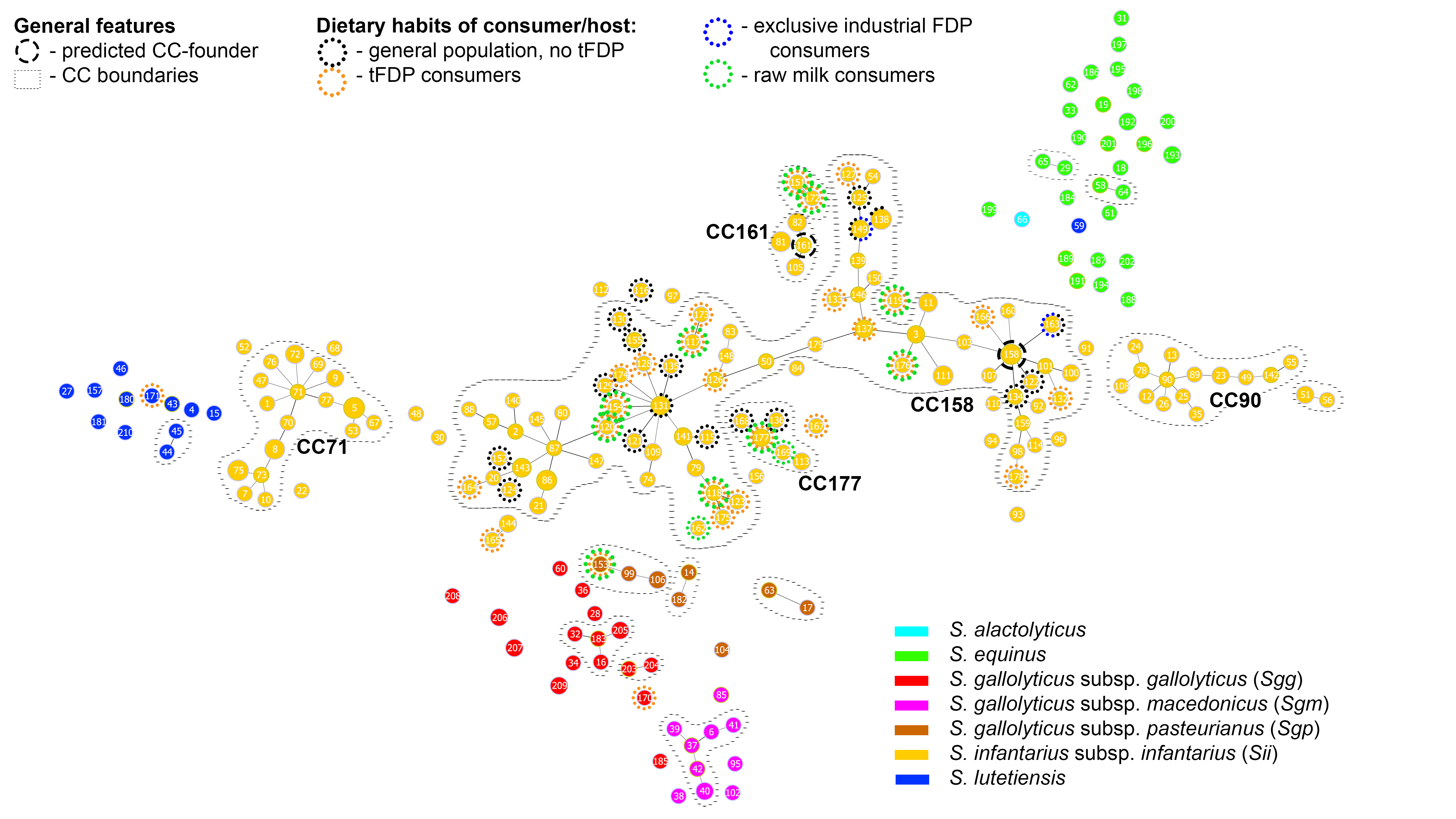

Supplement: S1 Fig — The tree is based on MLST profiles and colored according to SBSEC species with additional indications for CC founders and dietary habits of the host. (TIF) [file pone.0225452.s001.tif]
